# Editorial: Integrating imaging techniques to enhance precision in cancer immunotherapy

**DOI:** 10.3389/fonc.2026.1819541

**Published:** 2026-04-22

**Authors:** Jesus Pacheco-Torres

**Affiliations:** Institute for Biomedical Research Sols-Morreale (IIBM), CSIC-UAM, Madrid, Spain

**Keywords:** cancer immunotherapy, molecular imaging, MRI, precision oncology, tumor micreoenvironment (TME)

## 1 Opening context

Cancer immunotherapy has transformed oncology by enabling durable clinical responses across multiple tumor types. However, its clinical impact remains constrained by a persistent challenge: the limited ability to accurately stratify patients and to monitor therapeutic response in a timely and biologically meaningful manner. Current biomarkers—predominantly tissue-based and invasive—fail to capture the spatial and temporal heterogeneity of tumor–immune interactions, particularly under treatment-induced remodeling.

Imaging offers a complementary and potentially transformative solution. By enabling non-invasive, whole-body, and longitudinal interrogation of tumors, imaging can bridge the gap between underlying biology and clinical decision-making. Beyond anatomical assessment, advanced imaging approaches are increasingly capable of capturing functional, metabolic, and molecular features that reflect tumor behavior and immune dynamics.

This Research Topic was designed to address this translational need. It brings together studies that explore how diverse imaging techniques can enhance mechanistic understanding, improve biomarker development, and support more precise implementation of cancer immunotherapy.

## 2 The scientific rationale of the topic

The transition toward precision immunotherapy requires tools that can interrogate tumor biology *in vivo*, dynamically and non-invasively. Imaging is uniquely positioned to fulfill this role by integrating structural, functional, and molecular information across spatial scales.

Advances in multimodal imaging—including quantitative MRI, PET-based molecular imaging, and hybrid platforms—have expanded the capacity to characterize tumor heterogeneity, vascular function, cellular density, and immune-related processes. In parallel, computational approaches such as radiomics and machine learning are enabling the extraction of high-dimensional imaging features, moving beyond qualitative interpretation toward predictive modeling.

Importantly, the role of imaging is evolving from response assessment to mechanistic interrogation. In immunotherapy, biological changes often precede measurable alterations in tumor size, rendering conventional criteria insufficient. Imaging biomarkers that capture early microenvironmental changes, immune activation, or metabolic shifts are therefore critical.

This Research Topic reflects this paradigm shift. It situates imaging at the interface between tumor biology and therapeutic response, emphasizing its emerging role as a quantitative and integrative biomarker platform within the broader landscape of precision oncology.

## 3 Overview and synthesis of contributing articles

The four contributions included in this Research Topic collectively illustrate the diversification and convergence of imaging strategies aimed at improving precision in cancer immunotherapy. Rather than representing isolated approaches, these studies align along complementary axes: quantitative imaging, molecular interrogation, microenvironmental characterization, and data-driven integration.

### 3.1 Quantitative MRI as a dynamic biomarker of response

In Han et al., the authors investigate the use of T1 mapping and intravoxel incoherent motion (IVIM) MRI parameters to evaluate immunotherapy response in advanced lung cancer. Their findings show that post-treatment quantitative metrics—particularly reductions in T1 values and increases in diffusion-related parameters—differentiate responders from non-responders, with strong predictive performance (AUC values approaching 0.9). Importantly, these changes reflect microenvironmental alterations occurring during treatment, highlighting the ability of quantitative MRI to capture early biological response beyond conventional size-based criteria.

This work exemplifies a broader transition toward longitudinal, treatment-sensitive imaging biomarkers capable of informing therapeutic decisions.

### 3.2 Molecular imaging of therapeutic processes

A complementary perspective is provided by Rand et al., which leverages reporter gene imaging to directly visualize therapeutic delivery and activity. By incorporating the human sodium iodide symporter (hNIS) into oncolytic viral platforms, this approach enables real-time tracking of viral distribution and associated immune responses.

This strategy extends imaging beyond indirect inference toward direct interrogation of therapeutic mechanisms, enabling spatially resolved assessment of treatment engagement and heterogeneity.

### 3.3 Metabolic and microenvironmental imaging

At a finer biological scale, Cossa et al. discusses fluorescence lifetime imaging microscopy (FLIM) as a tool to probe cellular metabolism and tumor–immune interactions. By capturing endogenous metabolic signatures, FLIM provides insight into functional states associated with immune activation and tumor adaptation.

Although currently more established in preclinical settings, this work highlights the relevance of metabolic imaging as a proxy for immune function, complementing structural and functional modalities.

### 3.4 Emerging research trends and data-driven integration

Finally, Zhou et al. offers a bibliometric analysis that contextualizes these advances within the broader evolution of the field. The study identifies a rapid expansion of research at the intersection of imaging, immunotherapy, and computational analysis. As illustrated by keyword clustering analyses (Figure 6, page 9), radiomics, PET/CT, and artificial intelligence are emerging as central themes, reflecting a shift toward integrative and data-driven imaging frameworks.

This contribution underscores that the field is not only advancing technologically but also structurally, moving toward multimodal and computationally augmented paradigms.

### 3.5 Conceptual convergence

Across these contributions, several converging principles can be identified:

- Imaging is evolving from descriptive to quantitative and mechanistic
- Biomarkers are shifting from static to dynamic and longitudinal
- Approaches are transitioning from single-modality to multimodal integration
- Interpretation is moving toward computational and predictive frameworks

Together, these studies illustrate a coherent trajectory toward imaging-enabled precision immunotherapy.

## 4 Emerging themes and future directions

The contributions in this Research Topic highlight several priorities for future research.

First, multimodal integration will be essential to capture the complexity of tumor–immune interactions. Combining structural, functional, and molecular imaging modalities—potentially within unified analytical frameworks—will enhance biological interpretability and predictive power.

Second, quantitative imaging and artificial intelligence will play an increasingly central role. As reflected in current research trends, high-dimensional feature extraction and predictive modeling are enabling the development of robust imaging biomarkers.

Third, standardization and validation remain critical challenges. The translation of imaging biomarkers into clinical practice requires reproducibility across platforms, institutions, and patient populations.

Finally, clinical integration demands alignment with therapeutic decision-making. Imaging must not only reflect biological processes but also provide actionable information for patient stratification, treatment adaptation, and outcome prediction.

## 5 Concluding perspective

The integration of advanced imaging techniques into cancer immunotherapy represents a fundamental step toward achieving precision oncology. By linking tumor biology, immune dynamics, and therapeutic response, imaging has the potential to transform patient management from empirical to mechanism-informed strategies.

The studies presented in this Research Topic collectively demonstrate that this transition is already underway. From quantitative MRI to molecular and metabolic imaging, and from single-modality approaches to data-driven integration, the field is converging toward a unified objective: enabling non-invasive, dynamic, and clinically actionable biomarkers.

Realizing this potential will require continued interdisciplinary collaboration, methodological rigor, and a sustained focus on translational applicability. In this evolving landscape, imaging is not ancillary to immunotherapy—it is becoming integral to its precision.

